# Prevalence and factors affecting anxiety and depression among adult people with physical disabilities in Saudi Arabia: a cross-sectional study

**DOI:** 10.3389/fpsyg.2025.1517340

**Published:** 2025-07-11

**Authors:** Khalil A. Saleh, Waled A. M. Ahmed, Habib Alrashedi, Sameer A. Alkubati, Mokhtar Abdu Almoliky, Gamil Ghaleb Alrubaiee, Talal Ali Hussein Alqalah, Galal Faisal Albani, Adel Omar Laradhi, Meshal Hadi Altryfy

**Affiliations:** ^1^Department of Medical Surgical Nursing, College of Nursing, University of Ha’il, Hail, Saudi Arabia; ^2^Community Health Nursing Department, Faculty of Nursing, Al-Baha University, Al-Baha, Saudi Arabia; ^3^Department of Nursing, Faculty of Medicine and Health Sciences, Hodeida University, Al Hudaydah, Yemen; ^4^Department of Nursing, Faculty of Medicine and Health Sciences, Taiz University, Taizz, Yemen; ^5^Department of Community Health, College of Nursing, University of Ha’il, Hail, Saudi Arabia; ^6^Department of Community Health and Nutrition, Al-Razi University, Sanaa, Yemen; ^7^Nursing Division, Faculty of Medicine and Health Sciences, Sana’a University, Sanaa, Yemen; ^8^Department of Maternal and Child Health Nursing, College of Nursing, University of Ha’il, Ha’il, Saudi Arabia; ^9^Primary Care Nursing, The Executive Management of the Nursing in Hail Health Cluster, Ha’il, Saudi Arabia

**Keywords:** anxiety, depression, people with disabilities, disability, handicaps, Hail, Saudi Arabia

## Abstract

**Introduction:**

Anxiety and depression are among the major challenges that influence the health of individuals, especially those with disabilities. This study aimed to assess the level of anxiety and depression and the factors that affect them among adult individuals with physical disabilities in Hail, Saudi Arabia.

**Methods:**

A descriptive cross-sectional study recruited 155 adult individuals with physical disabilities in the Hail region of Saudi Arabia. From January to May 2024, data were collected from people with disabilities using an online validated questionnaire based on the satisfaction and the HADS. The survey was sent to people with physical disabilities through the responsible bodies after being given the required permissions. The data were collected using a self-reported questionnaire.

**Results:**

The research included individuals between 20 and 40 years old, nearly half of whom (47.7%) were women. More than half of the participants were under 25 years old, and 80% of them complained of physical disability, which was mild (62.6%) and due to congenital causes (61.9%). The study findings also showed that 61.8% of people with physical disabilities was satisfied with the provided healthcare services and 32.9 and 18% of them reported high levels of anxiety and depression, respectively. Anxiety was significantly associated with the educational level, degree of disability, presence of chronic illnesses, and parking availability *p* < 0.05. Depression was significantly associated with the degree, cause, period of disability, the ability to visit the hospital alone, and the priority in the waiting area (*p* < 0.05). These associations were uncertain in correlation linear regression analysis, since moderate disabilities were significant predictors of anxiety and depression while other factors were not significantly associated.

**Discussion:**

The majority of people with disabilities in Hail, Saudi Arabia complain of borderline to abnormal levels of anxiety compared with one-third of participants in the aspect of depression. Education, degree, type, cause of disability, and presence of chronic diseases had a significant impact on mental health among people with disabilities.

## Introduction

According to the World Health Organization (WHO), over 1.3 billion people, representing 16% of the world’s population, have some form of disability (World Health Organization [WHO], 2023). People with disabilities face additional challenges that may lead to increased levels of stress, anxiety, and depression. Physical limitations and negative societal attitudes may hinder their ability to participate in social activities, affecting personal relationships and community involvement. These barriers can result in social isolation, further diminishing quality of life and exacerbating depressive symptoms (Asdaq et al., 2024; Gayman et al., 2008; Yang, 2006). In recent years, there has been a growing recognition of the need to study the mental health of individuals with disabilities within a comprehensive framework. This includes considering the complex interplay of biological, psychological, and social factors—an approach known as the biopsychosocial model. Supporting this perspective is the International Classification of Functioning, Disability, and Health (ICF), developed by the WHO in 2001, which provides a structured framework for understanding disability and health in a holistic context (World Health Organization [WHO], 2007).

According to the ICF framework, an individual’s health is not limited to the presence of a disability or physical illness. It is also influenced by personal factors such as age and self-esteem (Taylor and Stanton, 2007), as well as environmental factors such as family support, access to health services, and community attitudes (Engel, 1977; Marmot and Wilkinson, 2005). The lack of social participation and poor psychosocial support are associated with higher levels of anxiety and depression and poorer quality of life among people with disabilities. This research is also informed by the causal agency theory (Shogren et al., 2017), which emphasizes self-determination and autonomy as important to psychological wellbeing, and the Family Systems Theory, which holds that family dynamics have a big influence on people’s mental health (Huang et al., 2022). Studies have emphasized the importance of promoting independence and social integration to improve psychological wellbeing and quality of life (González-Ortega et al., 2023; von Mackensen et al., 2012).

Anxiety and depression are two of the most common mental disorders in the world. According to the American Psychiatric Association [APA] (2013), anxiety is usually defined as excessive worry, nervousness, or fear that interferes with day-to-day functioning, whereas depression is characterized by ongoing sadness, a loss of interest or pleasure, and physical or cognitive symptoms like exhaustion and trouble focussing (American Psychiatric Association [APA], 2013). According to the World Health Organization, estimated 264 million people worldwide suffered from an anxiety disorder and 322 million from a depressive disorder in 2015, with prevalence rates of 3.6 and 4.4% respectively (de Paula et al., 2020; World Health Organization [WHO], 2017). Anxiety, depression, poor self-efficacy, and impaired physical activity among individuals with disability have been identified as an important factors that negatively affect QoL (Choi et al., 2019; Hu et al., 2022; Peters et al., 2019). Therefore, anxiety and depression were selected for investigation due to their high prevalence, clear impact on quality of life, and relevance to national mental health priorities.

Many different factors contribute to the increased suffering of people with disabilities in their daily lives and their ability to access healthcare services, such as gender, age, economic status, community awareness, and religion, which may play an important role in the level of anxiety and depression among people with disabilities. For the suffering of those with disabilities to be prevented or lessened, it is vitally important to determine the factors that contribute to it (Hsieh et al., 2020; Roy et al., 2023; World Health Organization [WHO], 2023).

The Saudi disability survey in 2017, reports that the proportion of Saudis with disabilities (mild, severe, and extreme) is 7.1% of the total population, which is considered one of the social and medical challenges (Yousef, 2019). According to the 2022 Population and Housing Census, there are (5.1%), or 1,349,585 individuals with disabilities in the Kingdom of Saudi Arabia, and 78% of households with disabilities struggle to involve themselves in society (Statistics GAf, 2023). Bindawas, in 2018, examined the national and regional prevalence rates of disability, types, and severity in Saudi Arabia survey; in the Hail region, there is about 11,044 (2.1%) people with disabilities were reported (Bindawas and Vennu, 2018). Depression and anxiety disorders are the fourth and sixth leading causes of disability in Saudi Arabia according to Saudi National Mental Health Survey (2020). But, these were based on a limited sample and are not generalizable (Al-Subaie et al., 2020). Furthermore, a recent study by Alnasser et al. (2024), showed that anxiety prevalence among general population was estimated to be 18% which was associated with physical or chronic conditions (Alnasser et al., 2024).

Many reasons may lead to disability. Genetic factors and traumatic accidents are among the most important causes in the Kingdom of Saudi Arabia (Albejaidi and Nair, 2019). The Kingdom of Saudi Arabia pays great attention to people with disabilities by enhancing education, health care, and community support, in addition to enabling them to access all health and service facilities and encouraging their integration into society in a way that enhances their physical and psychological health and quality of life. At the same time, the government has provided many programs that aim to prevent and reduce disability, as well as early detection of disability cases and providing treatment and rehabilitation (Alharbi et al., 2020; Malik et al., 2024; Mitchell and Alfuraih, 2018; Rahman and Qattan, 2021). This situation emphasizes the importance of paying great attention to people with disabilities to protect their wellbeing and safety.

People with disabilities may require continuous health care support, frequent medical check-ups, home-based assistance, and other supporting services, including equipment provision, which make the care programs quite costly (Iezzoni et al., 2021). Due to their diseases and a lack of adequate facilities, some people with disabilities must be transferred to nursing facilities. It may be necessary to provide specialized medical, social, psychological, vocational, and other rehabilitative care to manage persons with disabilities (Blunden, 2021). Emotional and social health is a crucial metric when gauging the general health and wellbeing of individuals from diverse backgrounds. In Saudi Arabia, a country undergoing rapid social and economic change, identifying the health conditions and health services of people with disabilities is critical for policy creation, healthcare provision, and societal engagement (Rahman and Qattan, 2021).

To the best of our knowledge, the previous studies on disabilities and their related aspects are limited globally, and in the Kingdom of Saudi Arabia. The investigation of the anxiety and depression among people with disabilities may contribute to a clear understanding of people with disabilities’ needs and policy creation and healthcare provision to such a population. Therefore, this study aimed to investigate the level of anxiety and depression among people with physical disabilities, their satisfaction with and access to healthcare services and to identify the factors affecting their psychological wellbeing in Saudi Arabia. This component was investigated to better understand how service availability and accessibility may influence psychological wellbeing among people with disabilities.

## Materials and methods

### Study design

A descriptive cross-sectional study was conducted among people with physical disabilities in Hail, Saudi Arabia, over 5 months from January to May 2024.

### Study population

All adults with disabilities in Hail, Saudi Arabia, with different kinds of disabilities (physical, hearing, and vision) were invited to participate in this study, excluding the people with intellectual disabilities and children. This exclusion was due to the nature of the data collection (self-administered surveys), which made it impractical to involve individuals with intellectual disabilities or children.

### Study setting

The study was conducted in the Hail region, which has an area of 128,588 km^2^ and is located in the northwest of Saudi Arabia. According to the last census, its population is about 732,000 people. The region is linked to eight governorates and about 100 canters are linked to the governorates.

### Sampling and sample size

The study employed convenience sampling to recruit people with physical disabilities. The sample size for the survey was 155 people with physical disabilities. A total of 250 individuals were invited to participate, and 168 completed the survey, 13 of them were excluded because they did not complete the survey, resulting in a response rate of 62%.

### Data collection technique and tools

Data was collected using an online survey. The survey was sent to people with physical disabilities after obtaining the required permission from the authorities caring for those people with physical disabilities and obtaining ethical approval. It was distributed under observation of the researcher and authorities caring for those people.

The questionnaire contained three parts; the first part contained the participants’ sociodemographic characteristics, such as age, gender, type, cause, and duration of disability. The second part measured satisfaction and access to healthcare services, using a tool adapted from previously used instruments in similar studies. It consists of 14 questions related to satisfaction level toward accessibility to the provided healthcare services. The tool used was scored as satisfied, not satisfied or not sure. The scale for the tool was distributed as 1 for satisfied and 0 for other options. The total of 14 points was calculated and categorized as satisfied (7–14 points), not satisfied (0–6 points).

The third part was the Hospital Anxiety and Depression Scale (HADS), the self-report rating scale comprising 14 items on a 4- 4-point Likert scale (range 0–3). It is designed to measure anxiety and depression (7 items for each subscale). The total score is the sum of the 14 items, and for each subscale, the score is the sum of the respective seven items (ranging from 0 to 21). A score of 0–7 is considered normal, 8–10 is a borderline case, and 11–21 is a case (anxiety or depression). (Zigmond and Snaith, 1983)

The HADS was developed by Zigmond and Snaith (1983) in 1983. Its purpose is to provide clinicians with an acceptable, reliable, valid, and easy-to-use practical tool for identifying and quantifying depression and anxiety symptoms. It presented high internal consistency; Cronbach’s α coefficient was 0.884 (0.829 for anxiety and 0.840 for depression) and stability (test-retest intraclass correlation coefficient 0.944). It was translated into the Arabic language by Terkawi et al. (2017). The version translated by Terkawi et al. (2017) was utilized and tested among individuals with disabilities in another region of the country. The results demonstrated that it is both valid and reliable, with acceptable levels of internal consistency. The Cronbach’s α for the HADS anxiety subscale was 0.83 (95% CI: 0.79–0.88), and for the HADS depression subscale, it was 0.77 (95% CI: 0.70–0.83).

### Data analysis

The data were analyzed using descriptive statistics such as frequencies and percentages in addition to mean and standard deviation for numerical variables such as anxiety and depression scores which were used to summarized the scores of numerical variables included in the our preliminary data, and inferential statistics such as One-way ANOVA and independent *t*-tests were used to determine the relationship between the study variables. HADS scores were treated as continuous variables in all inferential analyses in accordance with previous research. Multiple linear regression was used to determine the factors affecting anxiety and depression. Data were analyzed using the program SPSS 21.0 for Windows (SPSS, Inc., Chicago, IL) with statistical significance set at *P* < 0.05.

### Ethical approval

Ethical approval was obtained from the University of Hail Institutional Review Board (IRB Approval Number: H-2023-394, Date: October 30, 2023). The participants who were willing to participate received a cover letter explaining the purpose and outcomes of the study, and we assured them that their participation was voluntary, with the right to withdraw without any penalty at any time. Furthermore, ethical conduct was maintained during data collection and throughout the research process. The data will be kept strictly confidential.

## Results

### Sociodemographic characteristics of participants

Among 155 participants in this study, the participants were both males (52.3%) and females (47.7%), and those under 25 years of age were (53.5%); the majority of them (86.5%) lived in rural areas, and most of them were single. The educational level of 46.5% was secondary school education, and more than half were students. Regarding disability, most of them suffer from mild physical disability due to congenital causes since birth (62.6%), (61.9%), and (61.3%) respectively (Table 1).

**TABLE 1:** Demographic characteristics of the participants (*N* = 155).

Variable	Categories	Frequency n (%)
Age	<25 years	83 (53.5%)
	25–30 years	46 (29.7%)
	>30 years	26 (16.8%)
Gender	Male	81 (52.3%)
	Female	74 (47.7%)
Residence	Urban	134 (86.5%)
	Rural	21 (13.5%)
Marital status	Single	110 (71%)
	Married	39 (25.2%)
	Divorced	6 (3.9%)
Education	Secondary school	72 (46.5%)
	Diploma	37 (23.9%)
	Bachelor	44 (28.4%)
	Master	2 (1.3%)
Job	Student	89 (57.4%)
	Unemployed	28 (18.1%)
	Employed	38 (24.5%)
Type of disability	Physical	124 (80.0%)
	Hearing	16 (10.3%)
	Visual	15 (9.7%)
Degree of disability	Mild	97 (62.6%)
	Moderate	41 (26.5%)
	Severe	17 (11.0%)
Cause of disability	Congenital	96 (61.9%)
	Disease	24 (15.5%)
	Trauma	35 (22.6%)
Period of disability	Since Birth	95 (61.3%)
	<1 years	14 (9.0%)
	1–5 years	30 (19.4%)
	>5 years	16 (10.3%)

### Access to healthcare services and satisfaction levels

Overall satisfaction with healthcare services among participants was 61.8%. Most respondents had health insurance (78.1%) and reported receiving care from physicians and nurses positively. However, aspects of physical accessibility such as toilet accommodations and emergency response infrastructure were reported as insufficient (Table 2).

**TABLE 2:** Health care access and satisfaction of people with physical disabilities (*N* = 155).

Variable	Categories	Frequency n (%)
Health insurance	Yes	121 (78.1%)
	No	34 (21.9%)
Presence of chronic illness	Yes	27 (17.4%)
	No	128 (82.6%)
Visiting the hospital alone	Yes	105 (67.7%)
	No	50 (32.3%)
Waiting area: comfortable seats	Yes	123 (79.4%)
	No	32 (20.6%)
Waiting area: priority in waiting	Yes	93 (60.0%)
	No	47 (30.3%)
	Not Sure	15 (9.7%)
Toilets: only for people with disabilities	Yes	95 (61.3%)
	No	16 (10.3%)
	Not Sure	44 (28.4%)
Toilets: door opens outward	Yes	11 (7.1%)
	No	127 (81.9%)
	Not Sure	17 (11.0%)
Toilets: emergency button or phone	Yes	27 (17.4%)
	No	42 (27.1%)
	Not Sure	86 (55.5%)
Parking for people with disabilities	Yes	130 (83.9%)
	No	24 (15.5%)
	Not Sure	1 (0.6%)
Parking sufficiency	Yes	121 (78.1%)
	No	28 (18.1%)
	Not Sure	6 (3.9%)
Satisfaction to physicians	Satisfied	121 (78.1%)
	Not Satisfied	27 (17.4%)
	Not Sure	7 (4.5%)
Satisfaction to nurses	Satisfied	127 (81.9%)
	Not Satisfied	17 (11.0%)
	Not Sure	11 (7.1%)
Satisfaction to physiotherapist	Satisfied	101 (65.2%)
	Not Satisfied	18 (11.6%)
	Not Sure	36 (23.2%)
Satisfaction to reception staff	Satisfied	118 (76.1%)
	Not Satisfied	10 (6.5%)
	Not Sure	27 (17.4%)
Total satisfaction	Satisfied	61.8%
	Not satisfaction	38.2%

### Prevalence of anxiety and depression

**FIGURE 1:**
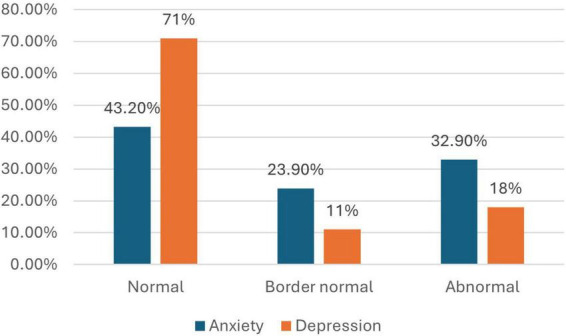
Prevalence of anxiety and depression among people with disabilities.

Among the participants, 32.9% reported high levels of anxiety, while 18% had high levels of depression. These findings highlight a significant burden of psychological distress among people with disabilities (Figure 1).

### The association of sociodemographic characteristics with the incidence of anxiety and depression

Statistical analysis revealed that education level had a significant association with anxiety scores (*p* = 0.03), where individuals with only secondary school education scored significantly higher in anxiety compared to those having a diploma or bachelor’s degree. In contrast, there was no statistical significance in the level of anxiety and depression scores with different ages, genders, residences, marital statuses, and jobs (*p* > 0.05) (Table 3).

**TABLE 3:** Association between the sociodemographic characteristics and anxiety and depression scores among people with physical disabilities (*N* = 155).

Characteristics	Anxiety		Depression	
	Mean ± SD	*P*-value	Mean ± SD	*P*-value
**Age**				
<25 years	8.61 ± 4.67	0.32	6.28 ± 3.95	0.115
25–30 years	8.65 ± 4.49		5.72 ± 3.15	
>30 years	10.12 ± 4.26		7.58 ± 3.34	
**Gender**				
Male	8.89 ± 4.72	0.97	6.15 ± 3.27	0.521
Female	8.86 ± 4.40		6.53 ± 4.06	
**Residence**				
Urban	8.89 ± 4.61	0.94	6.41 ± 3.82	0.486
Rural	8.81 ± 4.35		5.81 ± 2.42	
**Marital status**				
Single	9.01 ± 4.71	0.55	6.30 ± 3.80	0.848
Married	8.50 ± 4.13		6.43 ± 3.27	
**Education**				
Secondary school	9.64 ± 4.71	**0.03**	6.35 ± 3.51	0.687
Diploma	9.27 ± 4.59		6.70 ± 3.54	
Bachelor	7.37 ± 3.99		6.00 ± 4.01	
**Job**				
Employed	9.20 ± 4.74	0.127	6.50 ± 3.90	0.321
Unemployed	7.89 ± 3.84		5.82 ± 2.76	

### The association of health status, access, and satisfaction of people with physical disabilities with the incidence of anxiety and depression

The results showed that there was a significant association between the level of anxiety scores and degree of disability, presence of chronic diseases, and parking sufficiency (*p* < 0.001, 0.021, and 0.022, respectively). Other results showed a statistically significant level of depression scores and degree of disability, cause of disability, period of disability, visiting the hospital alone, and priority in the waiting area (*p* < 0.001, 0.024, 0.021, 0.048, and 0.045, respectively) (Tables 4, 5).

**TABLE 4:** Association between health status of people with physical disabilities with the overall anxiety and depression (*N* = 155).

Variables	Anxiety		Depression	
	Mean ± SD	*P*-value	Mean ± SD	*P*-value
**Type of disability**				
Physical	9.05 ± 4.72	0.508	6.31 ± 3.58	0.975
Visual	7.60 ± 3.96		6.53 ± 4.42	
Hearing	8.75 ± 3.71		6.31 ± 3.79	
**Degree of disability**				
Mild	7.65 ± 4.16	**0.001**	4.97 ± 2.67	*p* < 0.001
Moderate	10.44 ± 4.24		8.15 ± 4.06	
Severe	12.12 ± 4.97		9.71 ± 3.64	
**Cause of disability**				
Congenital	8.78 ± 4.70	0.326	5.76 ± 3.34	**0.024**
Disease	10.08 ± 3.99		7.92 ± 4.60	
Trauma	8.31 ± 4.50		6.80 ± 3.50	
**Period of disability**				
Since birth	8.76 ± 4.68	0.882	5.69 ± 3.38	**0.021**
< 1 yrs	9.29 ± 4.23		6.79 ± 3.14	
1–5 yrs	9.33 ± 4.48		6.97 ± 3.62	
> 5 yrs	8.38 ± 4.54		8.50 ± 4.82	
**Health insurance**				
Yes	9.20 ± 4.66	0.098	6.28 ± 3.42	0.759
No	7.74 ± 4.04		6.50 ± 4.45	
**Presence of chronic diseases**				
Yes	10.70 ± 4.60	**0.021**	7.48 ± 3.52	0.072
No	8.49 ± 4.47		6.09 ± 3.65	

Bold values represent statistically significant results at *p* < 0.05.

**TABLE 5:** Association between health care access, and satisfaction of people with physical disabilities with the overall anxiety and depression (*N* = 155).

Variables	Anxiety		Depression	
	Mean ± SD	*P*-value	Mean ± SD	*P*-value
**Health care access**				
Yes	9.00 ± 4.78	0.629	6.03 ± 3.27	0.139
No	8.62 ± 4.09		6.96 ± 4.34	
**Visiting the hospital alone**				
Yes	8.89 ± 4.44	0.963	6.03 ± 3.27	**0.048**
No	8.84 ± 5.06		7.47 ± 4.78	
**Priority in waiting area**				
Yes	8.86 ± 4.43	0.860	6.19 ± 3.35	**0.045**
No	9.44 ± 5.61		8.44 ± 4.93	
Not sure	8.70 ± 4.53		5.86 ± 3.62	
**Toilets: for people with disabilities**				
Yes	8.82 ± 5.40	0.985	7.64 ± 4.30	0.213
No	8.91 ± 4.49		6.37 ± 3.67	
Not sure	8.71 ± 4.77		5.18 ± 2.96	
**Parking sufficiency**				
Yes	9.18 ± 4.38	**0.022**	6.33 ± 3.51	0.477
No	8.61 ± 5.17		6.68 ± 4.53	
Not sure	4.00 ± 2.00		4.67 ± 1.21	
**Satisfaction to Physicians**				
Satisfied	9.02 ± 4.60	0.305	6.29 ± 3.66	0.968
Not satisfied	8.89 ± 4.52		6.48 ± 3.52	
Not sure	6.29 ± 3.77		6.43 ± 4.72	
**Satisfaction to nurses**				
Satisfied	8.94 ± 4.59	0.483	6.31 ± 3.60	0.915
Not satisfied	9.41 ± 4.32		6.65 ± 3.79	
Not sure	7.36 ± 4.72		6.09 ± 4.39	
**Satisfaction to physiotherapist**				
Satisfied	9.18 ± 4.76	0.273	6.27 ± 3.50	0.957
Not satisfied	9.33 ± 5.02		6.50 ± 3.82	
Not sure	7.81 ± 3.59		6.42 ± 4.09	

Bold values represent statistically significant results at *p* < 0.05.

### Multivariate analysis: predictors of anxiety and depression

A multiple linear regression was conducted to identify predictors of anxiety among people with physical disabilities. The model showed that individuals with moderate disabilities were significantly more likely to have higher anxiety scores compared to those with mild disabilities (β = −0.711; 95% CI: −0.992, −0.431; *p* < 0.001). Additionally, uncertainty about the availability of sufficient parking was also a significant predictor of increased anxiety (β = −0.928; 95% CI: −1.562, −0.295; *p* = 0.004). Although educational level and presence of chronic diseases were included in the model, they did not reach statistical significance (*p* > 0.05) (Table 6).

**TABLE 6:** Multiple linear regression of predictors of anxiety among people with physical disabilities.

Variable	Anxiety			
	β	95% CI for β		*P*-value
**Education**				
Secondary school	Reference			
Diploma	0.006	−0.299	0.312	0.968
Bachelor	−0.273	−0.558	0.012	0.060
**Degree of disability**				
Mild	Reference			
Moderate	−0.711	−0.992	−0.431	**0.000**
Severe	0.277	−0.167	0.720	0.219
**Presence of chronic diseases**				
Yes	Reference			
No	−0.285	−0.606	0.036	0.081
**Parking Sufficiency**				
Yes	Reference			
No	−0.234	−0.557	0.089	0.155
Not sure	−0.928	−1.562	−0.295	**0.004**

Bold values represent statistically significant results at *p* < 0.05.

Regarding depression, the analysis revealed that the severity of disability was a strong and significant predictor. Participants with moderate disabilities had higher depression scores (β = 0.507; 95% CI: 0.238, 0.776; *p* < 0.001), and those with severe disabilities had even higher scores (β = 1.037; 95% CI: 0.660, 1.414; *p* < 0.001), compared to those with mild disabilities. Other variables, such as cause and duration of disability, visiting the hospital alone, and waiting area priority, did not show significant associations with depression levels (Table 7).

**TABLE 7:** Multiple linear regression of predictors of depression among people with physical disabilities.

Variable	Depression			
	B	95% CI for β		*P*-value
**Degree of disability**				
Mild	Reference			
Moderate	0.507	0.238	0.776	p < 0.001
Severe	1.037	0.660	1.414	p < 0.001
**Cause of disability**				
Congenital	Reference			
Disease	0.463	−0.115	1.041	0.116
Trauma	0.063	−0.515	0.642	0.829
**Period of disability**				
From birth	Reference			
< 1 year	−0.021	−0.661	0.620	0.949
1–5 years	−0.060	−0.636	0.517	0.838
> 5 years	−0.096	−0.731	0.538	0.765
**Visiting hospital alone**				
Yes	Reference			
No	−0.016	−0.269	0.238	0.902
**Priority in waiting area**				
Yes	Reference			
No	0.063	−0.176	0.302	0.602

## Discussion

This study was the first study in Saudi Arabia to assess the anxiety and depression levels and associated factors among people with physical disabilities. Our study found a high rate of anxiety and depression among individuals with physical disabilities, which is in line with previous studies investigating these conditions among people with disabilities and it is crucial to explore the potential mechanisms contributing to these psychological outcomes. A key explanatory framework is the biopsychosocial model, which suggests that psychological wellbeing in individuals with disabilities is influenced by a dynamic interplay of biological limitations, psychological resilience, and social context. For example, as Mushtaq and Akhouri (2016) indicated individuals with disabilities typically have low self-esteem, which raises their anxiety and depressive symptoms. This situation may integrate both the effects of the disability and social attitudes toward people with disability, making those people feel low self-esteem and increasing vulnerability to developing anxiety and depression (Mushtaq and Akhouri, 2016). Compared to national estimates (Alnasser et al., 2024), this study showed more prevalent anxiety and depression appeared among people with disabilities, which is consistent with other research showing a link between psychological problems and physical disabilities (Dong et al., 2020; Wang et al., 2022; Zahra et al., 2022).

Similar findings were found in studies conducted on similar conditions; studies conducted on people with multiple sclerosis (MS) have also shown significant associations between physical disability and psychological issues. In a similar line to this study’s findings, a study conducted by Jones et al. (2014) reflected that people with disabilities were more likely to have anxiety and depression (Jones et al., 2014). The presence of chronic conditions such as MS, in addition to the physical disabilities of the people, may justify this association. The findings are consistent with several psychological theories that explain the psychiatric health challenges faced by individuals with severe physical disabilities. According to the causal agency theory emphasizes the role of autonomy in psychological wellbeing, suggesting that the inability to make independent decisions, such as attending medical appointments without assistance, may increase feelings of helplessness. Additionally, family systems theory highlights how inadequate or overly dependent family dynamics can exacerbate emotional distress (Huang et al., 2022; Shogren et al., 2017).

One notable area of interest is the protective role of physical activity, highlighted in a study by Battalio et al. (2020), individuals with disabilities who engaged in physical activity reported lower anxiety and depression scores (Battalio et al., 2020). In this study, people with severe disabilities may be unable to engage in physical activity, which could partly justify the higher rates of anxiety and depression scores among this group of people. This underscores the need for interventions to promote physical activity suitable for the abilities of people with disabilities, which could result in lowering psychiatric health issues.

Furthermore, intellectual disabilities are also associated with a higher incidence of anxiety and depression among people with disabilities; a meta-analysis by Maïano et al. (2018) reflected that youth with intellectual disabilities are at higher risk of having these types of psychological issues . However, this study focused on physically people with disabilities; the connection between intellectual impairments and psychological problems underscores the need for comprehensive psychological services that mitigate both physical and intellectual disabilities. Integrating mental health screening into routine healthcare for people with disabilities could be beneficial in early detection and appropriate management of anxiety and depression.

The association between physical disabilities and psychological issues is not limited to adults or young people. A study by Dong et al. (2020) indicated that older people’s depression and anxiety were strongly correlated with physical disabilities, leading to a decrease in both psychological and physical health (Dong et al., 2020). Our findings echo this, reinforcing the need for a lifespan approach to psychological care for people with disabilities, including proactive screening and ongoing mental health support at all age stages.

These associations were uncertain in correlation linear regression analysis, since moderate disabilities were significant associated with higher anxiety levels among people with disabilities while other factors were not significantly associated with anxiety. Furthermore, the linear regression indicates that moderate to severe degrees of disability were linked to higher depression score among people with physical disabilities in Hail, Saudi Arabia.

The low *p*-values observed in the bivariate analysis, particularly for the variables “visiting hospital alone,” “priority in the waiting area,” and “parking sufficiency,” suggest that individuals who responded affirmatively to these items—i.e., those who reported going to the hospital alone, having priority in the waiting area, and access to sufficient parking—had higher depression scores. This finding is counterintuitive, and importantly, the associations disappear in the multiple regression analysis, indicating that the initial correlations were likely due to collinearity with another variable, most likely the degree of disability severity.

However, the significant findings of this study, there are some limitations. The first limitation is that it was conducted on a small sample size and located only in the Hail region, which may limit the generalizability of the study findings. Another limitation is that the study was conducted through an online self-reported survey, which could have been associated with inappropriate responses or irrelevant participants. In addition, the HADS is a screening tool for symptoms of anxiety and depression, not a diagnostic instrument. Therefore, the results reflect psychological distress levels, not confirmed clinical diagnoses. Although the HADS provides generally used cut-off points for categorizing scores into “normal,” “borderline,” and “case” levels, these categories were established in a hospital outpatient context using a small validation sample. As a result, they may not be generalizable to people with physical impairments living in the community. The type of disability and the level of disability is indicated by participants during the survey and is not indicated by a physician or health care provider which limit the findings of this study. The last limitation is that using a cross-sectional design during a short period could limit the significance of the findings.

## Conclusion

This study highlights the significant mental health burden among individuals with physical disabilities in Hail, Saudi Arabia, with elevated levels of anxiety and depression, particularly among those with moderate to severe disabilities. Disability severity emerged as a key predictor of psychological distress, suggesting that as functional limitations increase, emotional vulnerability increases. Although some associations did not persist in multivariate analysis, our results suggest that certain healthcare-related stressors, such as visiting hospitals alone, prioritizing waiting areas, and inadequate parking, may contribute to higher depression scores. These factors may reflect broader issues related to accessibility, perceived support, and independence-elements known to influence psychological outcomes for individuals with disabilities. Current findings highlight the necessity of developing interventions that enhance the individuals’ psychological and physical health. Future research should examine how these interventions influence and how healthcare professionals could help to manage this issue. Additionally, longitudinal studies are advised to determine the effects of physical disability over an extended period and its association with psychiatric disorders like depression and anxiety.

## Data Availability

The raw data supporting the conclusions of this article will be made available by the authors, without undue reservation.
